# *Lactobacillus paracasei* BD5115-Derived 2-Hydroxy-3-Methylbutyric Acid Promotes Intestinal Epithelial Cells Proliferation by Upregulating the *MYC* Signaling Pathway

**DOI:** 10.3389/fnut.2022.799053

**Published:** 2022-03-17

**Authors:** Zhenyi Qiao, Xiaohua Wang, Chaoyue Wang, Jin Han, Weiwei Qi, Huanchang Zhang, Zhenmin Liu, Chunping You

**Affiliations:** ^1^State Key Laboratory of Dairy Biotechnology, Shanghai Engineering Research Center of Dairy Biotechnology, Dairy Research Institute, Bright Dairy & Food Co., Ltd., Shanghai, China; ^2^State Key Laboratory of Dairy Biotechnology, Shanghai Engineering Research Center of Dairy Biotechnology, Postdoctoral Workstation of Bright Dairy–Shanghai Jiao Tong University, Dairy Research Institute, Bright Dairy & Food Co., Ltd., Shanghai, China; ^3^College of Food Science and Technology, Shanghai Ocean University, Shanghai, China; ^4^Shanghai Key Laboratory of Bio-Energy Crops, School of Life Sciences, Shanghai University, Shanghai, China

**Keywords:** 2-hydroxy-3-methylbutyric acid, transcription factor, colitis-associated cancer (CAC), *Lactobacillus paracasei* BD5115, intestinal epithelial cells proliferation

## Abstract

Metabolites of probiotics that are beneficial to human health have been isolated from the intestinal tract and natural dairy products. However, many studies on probiotics and prebiotics are limited to the observation of human cohorts and animal phenotypes. The molecular mechanisms by which metabolites of probiotics regulate health are still need further exploration. In this work, we isolated a strain of *Lactobacillus Paracasei* from human milk samples. We numbered it as *Lactobacillus Paracasei* BD5115. The mouse model of high-fat diet confirmed that the metabolites of this strain also promotes intestinal epithelial cells (IECs) proliferation. Single-cell sequencing showed that a bZIP transcription factor *MAFF* was specifically expressed in some IECs. We found that MAFF interacted with MBP1 to regulate the expression of *MYC*. Analysis of the active components in BD5115 metabolites confirmed that 2-hydroxy-3-methylbutyric acid promotes the expression of the *MYC* gene. This promotes the proliferation of IECs. Our findings indicate that 2-hydroxy-3-methylbutyric acid regulate *MYC* gene expression mediated by MAFF/MBP1 interaction. This study not only screened a strain with promoted IECs proliferation, but also discovered a new signal pathway that regulates *MYC* gene expression.

## Introduction

Oral microorganisms have attracted attention for being healthy for humans for a long time. In this case, the concept of probiotics and prebiotics first appeared in 1974. Probiotics have become an important commercial industry and the most frequently used food supplement in the world (1). Edible probiotics have been widely supported by many physicians and pediatricians, especially gastroenterologists, upon making clinical diagnoses (2, 3).

Improving the function of the gut barrier is a potential mechanism by which probiotics improve human health. These effects include upregulation of tight junction (TJ) proteins, promotion of mucus secretion, increased butyric acid levels and microbiome regulation (4, 5). These effects may be mediated by metabolites secreted by probiotics. These metabolites bind to receptors on the membrane of IECs, and signals are then transferred to the cells. This phenomenon is sufficient to trigger cascade reactions within cells, allowing IECs to respond to external conditions. For example, two proteins (P40 and p75) secreted by *Lactobacillus rhamnosus* GG (LGG) are thought to promote the stability of IECs by inhibiting the apoptosis of cytokine-related epidermal cells (6). However, due to the technical complexity of metabolite isolation and identification, considerable limitations remain, and probiotic health research still relies on only the evidence-based validation of clinical cohorts (7–12). This causes uncertainty regarding the industrialization of probiotics for use in food and clinical medicine.

We herein aimed to screen for probiotics or prebiotics that are capable of promoting IECs proliferation from our existing bacterial library through a comprehensive probiotic function screening platform and to study their specific molecular mechanism. We hoped to identify new probiotics that have health-promoting effects. We screened a *Lactobacillus paracasei* BD5115 strain that we originally isolated from breast milk samples using a Caco-2 cell model. The mouse model with high-fat diet (HFD) confirmed that BD5115 metabolites promote IECs proliferation. In addition, single-cell sequencing analysis showed that BD5115 metabolites promoted the proliferation of IECs. Among them, we found a bZIP transcription factor, *MAFF*, that was specifically highly expressed in the IECs of the BD5115 group. We found that this transcription factor interacted with *c-myc* promoter binding protein 1 (MBP1) and combined with the *MYC* promoter, which promoted the expression of the *MYC* gene in IECs. This is a newly identified signaling pathway that regulates the expression of the *MYC* gene. Analysis of the active components of BD5115 metabolites revealed that 2-hydroxy-3-methylbutyric acid (HMBA) was significantly enriched, and the expression of the *MYC* gene in Caco-2 cells was increased significantly after treatment with HMBA.

## Materials and Methods

### Screening of the Strains

In this study, 54 strains of different genera were selected from the bacterial library of our laboratory. These bacteria are classified as *Lactobacillus paracasei, Lactobacillus casei, Streptococcus thermophilus, Lactobacillus acidophilus, Lactobacillus bulgaricus, Lactobacillus salivarius, Paenibacillus bovis, Paenibacillus polymyxa, Lactobacillus rhamnosus* and *Lactobacillus bulgaricus* (Supplementary Table S1). These bacteria were isolated from different media and regions in this experiment, and they grew in skimmed milk. We cultured these bacteria in 10% skimmed milk for 24 h to obtain their metabolites. The metabolites were incubated with Caco-2 cells for 24 h, and the RNA from the Caco-2 cells was then extracted for mRNA sequencing. Because of the large number of bacteria, we carried out the experiments in two batches. To ensure the consistency of the data obtained from the two experiments, we established a negative control (NC) group in each batch. For the culture method of Caco-2 cells, refer to the cell culture section in materials and methods.

### Bacterial Strain and Cultivation

*L. paracasei* BD5115 (CGMCC No. 20045) was provided by the State Key Laboratory of Dairy Biotechnology, Shanghai 200436, China. The bacterial strain was routinely cultivated aerobically on milk agar at 30°C for 24 h. The medium was prepared by adding 10 ml of sterile 10% (w/w) reconstituted skim milk to 100 ml of melted 1.5% (v/w) agar solution.

### Preparation of the BD5115 Metabolites

The lyophilized fermentation product powder was prepared as following steps. Briefly, a loop of freshly cultivated BD5115 on milk agar was inoculated into a 100 ml flask containing 20 ml of sterile 10% (w/w) reconstituted skim milk and cultivated at 30°C at 180 rpm for 24 h. The culture was then transferred at a ratio of 4% (v/v) to a 250 ml flask containing 50 ml sterile 10% (w/w) reconstituted skim milk and cultivated at 30°C for 72 h. The bacterial culture was centrifuged at 8,000 × g at 4°C for 10 min to remove bacterial cells, and the supernatant was lyophilized under vacuum. The lyophilized metabolite preparation was then stored at −80°C.

### Dosage Information

Because metabolites and not bacteria were used in our experiment, we converted colony-forming units (CFU) to metabolite weight. In our system, ~7 × 10^10^ CFU of bacteria and approximately 50 g of metabolites were cultivated in 1 L of medium. We calculated a maximum dose of 10^10^ CFU for healthy adult humans (60 kg). Taking into account the human-to-mouse dose conversion, the equivalent mice dose is 10^9^ CFU/kg. Since an 16-week-old mouse weighs no more than 50 g, the equivalent daily gavage dose for a mouse is 5 × 10^8^ CFU. Therefore, the appropriate daily dose is 0.375 g/kg/d (8 mL/kg/d) of metabolites. In addition, since we cultured BD5115 with 10% skimmed milk (dissolved in purified water), we also used 10% skimmed milk as the vehicle treatment.

### Cell Culture

The Caco-2 cell line was cultured in DMEM (GE Healthcare, SH30021.01) supplemented with 10% FBS (GE Healthcare, SH30396.03). Caco-2 cells were cultured in 96-well plates in a humidified incubator at 37°C with 5% CO_2_ for 4 days. The digested Caco-2 cells were plated at a density of 1.5 × 10^5^ cells per well, and the BD5115 metabolites were added to the wells at concentrations no >5% (v/v). The mixture was incubated for an additional 4 h and centrifuged to remove the supernatant.

### High-Fat-Diet Feeding

The animal experiments including HFD and CAC mice were approved and authorized by the Shanghai Institute of Family Planning (identification number 2020-34 “Effect of probiotics on body weight and body fat”). The 8-week-old male mice (C57BL/6 mice) were subjected to a constant 12 h day–night cycle and a constant room temperature of 22°C. A total of 30 mice were randomly divided into three groups. Ten mice were fed a normal chow diet (NCD) consisting of 70 kJ% carbohydrates, 22 kJ% proteins and 5 kJ% fat and the other twenty mice fed a HFD consisting of 20 kJ% carbohydrates, 20 kJ% proteins and 60 kJ% fat. The mice had excess to food and water. After 8 weeks of feeding on a HFD, half of HFD mice were administered with BD5115 metabolites for another 4 weeks. As a control, the other mice were administered skimmed milk. HFD was still maintained during the period. In the insulin tolerance test (ITT), we first fasted the mice for 6 h. Adequate drinking water is provided during this period. In total 0.5U/kg insulin was injected into mice by intraperitoneal injection. Blood glucose levels were measured at 0, 15, 30, 60, and 120 min. For organ collection, the mice were sacrificed by cervical dislocation or CO_2_ inhalation. We use the median with range method for statistical analysis, and use the TTEST method to analyze the significance. We use GraphPad for graphing.

### Colitis-Associated Cancer (CAC) Induction Using AOM/1.5% DSS

According to previous works, different strains of mice may have different sensitivity and tolerance to AOM (Azoxymethane) / DSS (Dextran Sulfate Sodium Salt) due to genetic factors (13). Azoxymethane (AOM)/ Dextran sodium sulfate (DSS) mouse model is an animal model that develops from inflammatory bowel disease (IBD) to colorectal cancer (14). AOM/DSS model can well simulate the physiological and pathological process of cancer induced by chronic intestinal inflammation, and has been widely used to study the mechanism of cancer formation in recent years. C57BL/6J mice have lower tolerance to DSS, which leads to longer duration of inflammation and easier induction into mice with CAC (15, 16). Moreover, although Snider et.al believe that mice of different sexes are vulnerable to AOM/DSS, the experimental results of female mice are easier to be repeated (17–19). In addition, it has been reported that estrogen may also promote the progression of enteritis related tumors. Therefore, we selected female C57BL/6J mice for the experiment. Colorectal cancer was induced by AOM and 1.5% DSS. For CAC induction, only female mice aged 8 weeks were used. A total of 20 mice were randomly divided into two groups. Briefly, 8-week-old females (NCD-fed C57BL/6 mice) were injected with 10 mg/kg AOM (A5486, Sigma-Aldrich) on day 1 of the protocol. On day 5, the mice were exposed to 1.5% DSS (MW036000-50000, 0216011080, MP Biomedicals) via their drinking water for 5 d, with one intermediate exchange of DSS-containing water. A minimum of 5 mL of DSS solution was provided to each mouse per day. The colitis test lasts 10 days. On day 10, the drinking water was replaced with normal drinking water until the end of the experiment. At day 62, tumor-bearing mice were euthanized to obtain non-tumor and tumor tissues. The tumors were counted in a blinded fashion. We use the median with range method for statistical analysis, and use the TTEST method to analyze the significance. We use GraphPad for graphing.

### H&E Staining

Abcam's H&E staining kit was used in the dyeing process according to the manufacturer's instructions. In brief, an adequate amount of hematoxylin (Mayer's, Lillie's Modification) to completely cover the tissue sections was applied for 5 mins. The slides were rinsed twice with distilled water to remove excess stain. An adequate amount of Bluing Reagent was applied to completely cover the tissue sections for 10–15 secs. The slides were rinsed twice with distilled water and then dipped in in absolute alcohol; excess alcohol was removed by blotting. An adequate amount of eosin solution (modified alcohol) was applied to completely cover the tissue sections, and the sections were incubated for 2–3 mins. The slides were rinsed with absolute alcohol. The slides were dehydrated with absolute alcohol three times. The slides were cleared and mounted with synthetic resin.

### Transmission Electron Microscopy

The colon tissues were soaked in paraformaldehyde and postfixed in osmium tetraoxide. Fixed samples were dehydrated with an ethanol gradient up to 100% and then transferred into a propylene oxide solution and slowly embedded in acrylic resin (London Resin Company). Thin sections (70 nm) were sliced using a diamond knife microtome (Reichert Ultracut E). The sections were placed on 100-mesh copper grids and stained with uranyl acetate for 30 min and with lead citrate for 15 min. The sections were observed with a transmission electron microscope (Hitachi H7600).

### Single-Cell RNA Sequencing

During the sample preparation process, we collected ~1 cm of the proximal colon tissues from HFD+SM and HFD+BD5115 group, removed the mesentery, and recovered the content of the colon segment using PBS. The tissue was dissociated into a single-cell suspension by enzyme digestion. Briefly, the tissues were cut into approximately 1 mm^2^ pieces and digested using the Solo^TM^ Tumor Dissociation Kit (JZ-SC-58201) at 37°C for 50 min. After stopping digestion by the addition of excess DMEM, the cell strainer-filtered single-cell solution was kept on ice until it was loaded into a BD Rhapsody cartridge for single-cell transcriptome isolation.

Based on the BD Rhapsody system whole-transcriptome analysis alpha protocol for single-cell whole-transcriptome analysis, microbead-captured single-cell transcriptomes were used to prepare a cDNA library containing cell label and UMI information. Briefly, double-stranded cDNA was first generated from the microbead-captured single-cell transcriptome in several steps, including reverse transcription, second-strand synthesis, end preparation, adapter ligation, and whole-transcriptome amplification. Then, the final cDNA library was generated from double-stranded full-length cDNA by random priming amplification using a BD Rhapsody cDNA Kit (BD Biosciences, 633773) and the BD Rhapsody Targeted mRNA and AbSeq Amplification Kit (BD Biosciences, 633774). The library was sequenced in PE150 mode (paired-end with 150-bp reads) on an X Ten instrument (Illumina).

Raw reads were processed through the BD Rhapsody Whole-Transcriptome Assay Analysis Pipeline (early access); the processing included filtering by read quality, annotation of reads, annotation of molecules, determination of putative cells, and generation of a single-cell expression matrix. Briefly, read pairs with low sequencing quality (too long, too short, low sequencing score or high single-nucleotide frequency) were first removed at the read quality filtering step. The quality-filtered R1 reads were analyzed to identify the cell label sequence (CL), the molecular identifier sequence (UMI), and the poly- dT tail sequence, and the quality-filtered R2 reads were mapped using STAR (version 2.5.2b) at the read annotation step. Further adjustments were performed using recursive substitution error correction (RSEC) and distribution-based error correction (DBEC) algorithms to remove artifactual molecules arising from amplification bias at the molecule annotation step. Putative cells were distinguished from background noise through a second derivative analysis at the putative cell determination step. Finally, putative cell information was combined with RSEC/DBEC-adjusted molecules to generate a single-cell expression matrix. The pipeline output provided raw gene expression matrices corrected by the RSEC and DBEC algorithms. Among all the matrices, UMI counts per cell corrected by the DBEC algorithm were later used in the clustering analysis.

Raw gene expression matrices from two cartridges were read separately into R (version 3.6.0) and converted to Seurat objects using the Seurat R package (version 3.0.1). CCA integration between two batches was performed with the Seurat R package.

The gene expression matrix was then normalized to the total cellular UMI count. The top 2,000 features were selected as highly variable genes for further clustering analysis. After scaling the data with respect to UMI counts, PCA was performed based on the highly variable genes identified in the previous step to reduce dimensionality. In addition, the first 50 principal components were chosen based on the PC heat map, jackstraw plot, and PC elbow plot to further reduce dimensionality using the tSNE algorithm. Clusters were identified with the default setting using the RunTSNE function. Each cluster was then annotated with canonical cluster markers.

Downstream pseudotime trajectory analysis was performed with the Monocle 2 R package.

### Co-immunoprecipitation

Caco-2 cells were cultured at a constant temperature for 72 h and lysed in RIPA buffer (R0278, Sigma Aldrich) on ice for 5 min. The lysate was then centrifuged at 12,000 rpm for 5 min, after which the pellet was discarded, and the sample was centrifuged one more time. For co-immunoprecipitation, the protein lysate was incubated with 2 μL of a rabbit MAFF antibody (M8194, Sigma Aldrich) for at least 1 h. Then, the protein-antibody complex was incubated with 50 μL of Protein-A Agarose (P2545, Sigma Aldrich) for at least 30 min. After washing with RIPA buffer 5 times, western blot was performed with the MAFF (1:1000 dilution) and MBP1 (1:1000 dilution) antibodies.

### RNA Extraction and Quantitative PCR

Total RNA was extracted from Caco-2 cells using an RNA extraction kit (DP430, TIANGEN), and cDNA was synthesized by a reverse transcription kit (KR123, TIANGEN). All of the primer pairs for quantitative PCR were designed using BLAST-Primer software (https://www.ncbi.nlm.nih.gov/tools/primer-blast/). The *CANX* gene (Ensembl Number: ENSG00000127022) was used as an internal control. For quantitative PCR, the reaction mixture comprised the cDNA first-strand template, primer mix and SYBR Green Mix (208054, QIAGEN) in a final volume of 10 μL. The reactions were performed using a QuantStudio 3 (Thermo Fisher Scientific). All experiments were repeated 3 times. The data were analyzed by the ΔΔCt method. The primers sequences are shown in Supplementary Table S6. We use the Mean ± SEM method for statistical analysis, and use the TTEST method to analyze the significance. We use GraphPad for graphing.

### mRNA Sequencing Analysis

Total RNA (10 μg) was isolated using an RNeasy mini kit (Qiagen, 74104). Paired-end libraries were synthesized using the TruSeq^TM^ RNA Sample Preparation Kit (Illumina, RS-122-2001) according to the instructions provided in the TruSeq^TM^ RNA Sample Preparation Guide. Briefly, poly-A-containing mRNA molecules were purified using magnetic beads attached to poly-T oligonucleotides.

Following purification, the mRNA was fragmented into small pieces using divalent cations at 94°C for 8 min. The cleaved RNA fragments were copied into first-strand cDNA using reverse transcriptase and random primers. This was followed by second- strand cDNA synthesis using DNA polymerase I and RNase H. These cDNA fragments were then subjected to an end repair process and the addition of a single “A” base, followed by ligation to the adapters. The products were then purified and amplified by PCR to create the final cDNA library. The purified libraries were quantified in a Qubit 2.0 fluorometer (Life Technologies, USA) and validated using an Agilent 2,100 Bioanalyzer (Agilent Technologies, USA) to confirm the insert size and calculate the molar concentration. Clustering was performed by cBot with the library diluted to 10 pM, and the library was then sequenced on the Illumina HiSeq platform (Illumina, USA). Differentially expressed genes were defined as those with fold changes >2 or <0.5 and *P* < 0.05.

Gene ontology is a huge database with a large amount of gene function information (http://geneontology.org). We used this database to cluster the functions of genes. The specific parameters are: Test Type: Fisher's exact; Correction: Calculate False Discovery Rate.

In the heat map analysis of differentially expressed genes, we used heml software (http://hemi.biocuckoo.org/down.php) for analysis.

### Electrophoretic Mobility Shift Assay (EMSA)

The His-MAFF and GST-MBP1 fusion proteins were expressed *in vitro* and used for EMSA.

Oligonucleotide probes were synthesized and labeled according to the standard protocol by Sangon Biotech (Shanghai) Co., Ltd. The standard reaction mixture for EMSA contained 20 ng of the purified His-MAFF or GST-MBP1 fusion protein, 5 ng of biotin-labeled annealed oligonucleotides, 2 μL of 10 × binding buffer (100 mM Tris, 500 mM KCl, and 10 mM DTT, pH 7.5), 1 mL of 50% (v/v) glycerol, 1 mL of 100 mM MgCl_2_, 1 mL of 1 mg/mL poly (dI-dC), 1 mL of 1% (v/v) Nonidet P-40, and double-distilled water to a final volume of 20 mL. The reactions were incubated at 25°C for 20 min, electrophoresed on 8% (w/v) polyacrylamide gels, and then transferred to N+ nylon membranes (Millipore). Biotin-labeled DNA was detected using the LightShift Chemiluminescent EMSA kit (20148, Thermo Fisher Scientific). Signals were visualized by X-ray film exposure. The probes sequences are shown in Supplementary Table S7.

### Transient Assays for the Detection of Activation Activity *in vivo*

The reporters were constructed based on the pMCS-*Cypridina*-LUC vector, and the effectors were constructed based on the pTricer^TM^ EF A Mammalian expression system. To generate pMCS *Pro:MYC*-*Cypridina*-LUC for dual-luciferase assays, the−300 bp promoter fragments from the TSSs of different *MYC* genes were amplified from Caco-2 cell genomic DNA by PCR.

To construct pTricer-*MAFF* and pTricer-*MBP1*, the ORFs of *MAFF* and *MBP1* were cloned into the pTricer^TM^ EF A Mammalian expression system.

Lipofectamine^TM^ 3000 Reagent (L3000001, Thermo Fisher Scientific) was used to transfect DNA into Caco-2 cells as described in the manufacturer's protocol. Transient dual-luciferase activity in Caco-2 cells was assayed and measured using a *Cypridina*-Firefly Luciferase Dual Assay Kit (16183, Thermo Fisher Scientific Pierce) as described in the protocol.

### Untargeted Metabolomics Analysis

We entrusted Shanghai Applied Protein Technology Company to carry out this experiment. Generally speaking, BD5115 metabolites samples were collected in 5 mL Vacutainer tubes containing the chelating agent ethylene diamine tetraacetic acid (EDTA), then the samples were centrifuged for 15 min (1500 g, 4°C). Each aliquot (150 μL) of the plasma sample was stored at −80°C until UPLC-Q-TOF/MS analysis. The plasma samples were thawed at 4°C and 100 μL aliquots were mixed with 400 μL of cold methanol/acetonitrile (1:1, v/v) to remove the protein. The mixture was centrifuged for 15 min (14000 g, 4°C). The supernatant was dried in a vacuum centrifuge. For LC-MS analysis, the samples were re-dissolved in 100 μL acetonitrile/water (1:1, v/v) solvent. To monitor the stability and repeatability of instrument analysis, quality control (QC) samples were prepared by pooling 10 μL of each sample and analyzed together with the other samples. The QC samples were inserted regularly and analyzed in every 5 samples.

After obtaining the original data, we use the orthogonal partial least squares discrimination analysis (OPLS-DA) method based on R language to analyze the original data to obtain the variable importance for the project (VIP). In metabonomics, OPLS-DA VI*P* > 1 and *P* value < 0.05 are usually used as the screening criteria for metabolites with significant differences, which is used as the screening criteria in this experiment. Finally, the changes of metabolic difference multiples were visually displayed by histogram.

### Flow Cytometry

Caco-2 cells treated with HMBA for 24 h were harvested by trypsin digestion. Then, the cells were incubated with fluorescein isothiocyanate (FITC) and propidium iodide (PI) from an Annexin V-FITC apoptosis detection kit (APOAF, Sigma-Aldrich). Then, the cells were detected by flow cytometry.

### TCGA Database

GEPIA (http://gepia.cancer-pku.cn/index.html) is a developed interactive web server for analyzing the RNA sequencing expression data of 9,736 tumors and 8,587 normal samples from the TCGA and the GTEx projects, using a standard processing pipeline. The specific analysis parameters are: Group cutoff: median; Hazards Ratio (HR): Yes; 95% Confidence Interval: Yes.

### Statistical Analysis

Statistical analysis was performed using Prism 6 (Graphpad). Data are plotted in the figures as median with range or mean ± SEM. Before *t-*test, D'Agostino & Pearson omnibus normality test was used to analyze the normal distribution of the data. Furthermore, in the process of equal variables analysis, we require *P* > 0.1. This ensures that all data meet statistical requirements. Differences between two treatment groups were assessed using two-tailed, unpaired Student's *t* test. Differences among three or four groups (glucose level, serum insulin, HOMA-IR and transactivation assay) were assessed using one-way ANOVA with Bonferroni *post-hoc* test. Two-way repeated-measures ANOVA with Bonferroni *post-hoc* test was used for analysis of ITT data. Significant differences are indicated in the figures by ^*^p < 0.05, ^**^p < 0.01, ^***^p < 0.001, ^****^p < 0.0001. Notable non-significant differences are indicated in the figures by “n.s.”.

## Results

### Effect of Bacterial Metabolites on Caco-2 Cell Gene Expression as Determined by mRNA Sequencing Analysis

Although many studies have reported that probiotics and prebiotics can promote the stability of IECs (20–23), few probiotics have been confirmed to regulate the gut barrier through systematic biological experiments and detailed molecular research.

**Figure 1:**
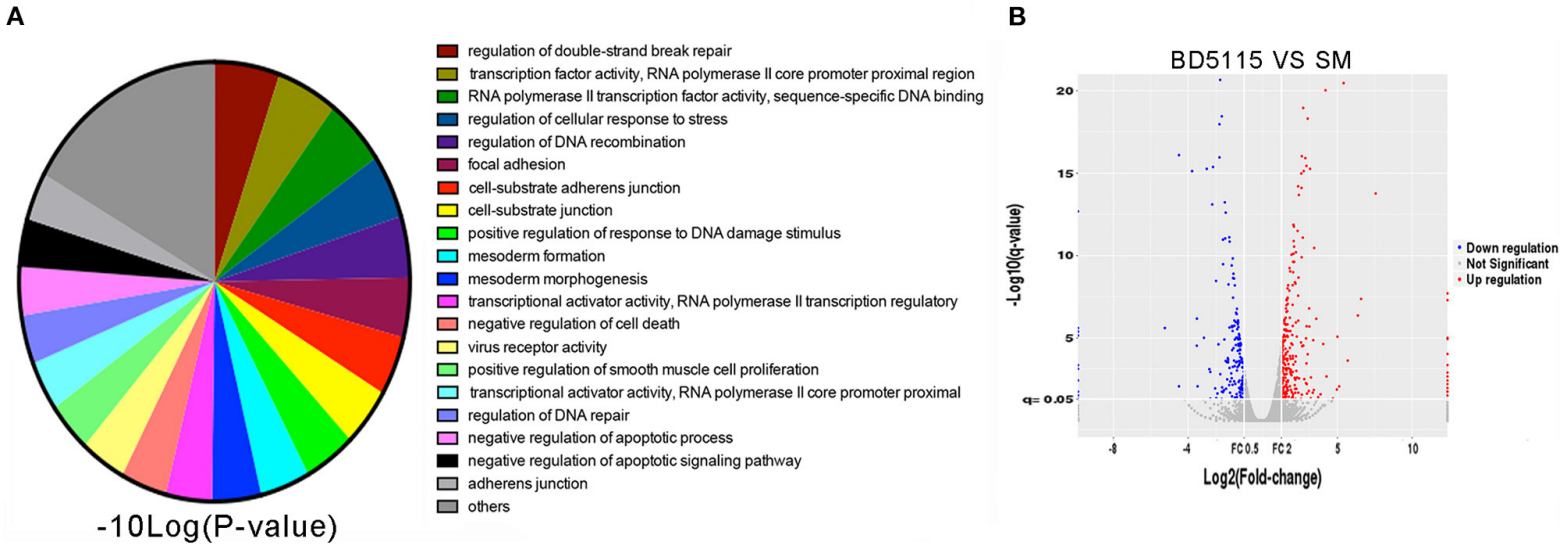
RNA sequencing screening. **(A)** Gene Ontology analysis of genes with altered expression in Caco-2 cells after treatment with BD5115 metabolites. The significance and number of genes classified within each GO term are shown. **(B)** Volcano map analysis of genes with altered expression in Caco-2 cells after treatment with BD5115 metabolites. The screening criteria for differentially expressed genes were a fold change >2 or <0.5 and *P* < 0.05.

In this study, 54 strains of different genera were selected from the bacterial library of our laboratory. We used Caco-2 cell line as our screening model to observe the regulatory effect of these bacteria metabolites in skim milk on Caco-2 cells. We also used RNA sequencing method to analyze gene expression in Caco-2 cells. Principal component analysis (PCA) of the RNA sequencing results revealed that both the NC samples were clustered in similar locations (Supplementary Figure S1). The results showed that the two RNA datasets were highly consistent. Then, each sample was subjected to Gene Ontology (GO) analysis. We found that the metabolites produced by *L. paracasei* BD5115 significantly altered the gene transcription, DNA repair and adhesion functions of Caco-2 cells (Figure 1A). Among them, 173 genes were upregulated, and 141 genes were downregulated (Figure 1B).

*L. paracasei* BD5115 is a bacterium we isolated from milk samples from the Tianmu Lake area, Jiangsu Province, China, in 2019. In our bacterial library, it is numbered BD5115. Therefore, we named the strain BD5115.

### Metabolites of *L. paracasei* BD5115 Alleviate HFD-Induced Hyperglycemia

**Figure 2:**
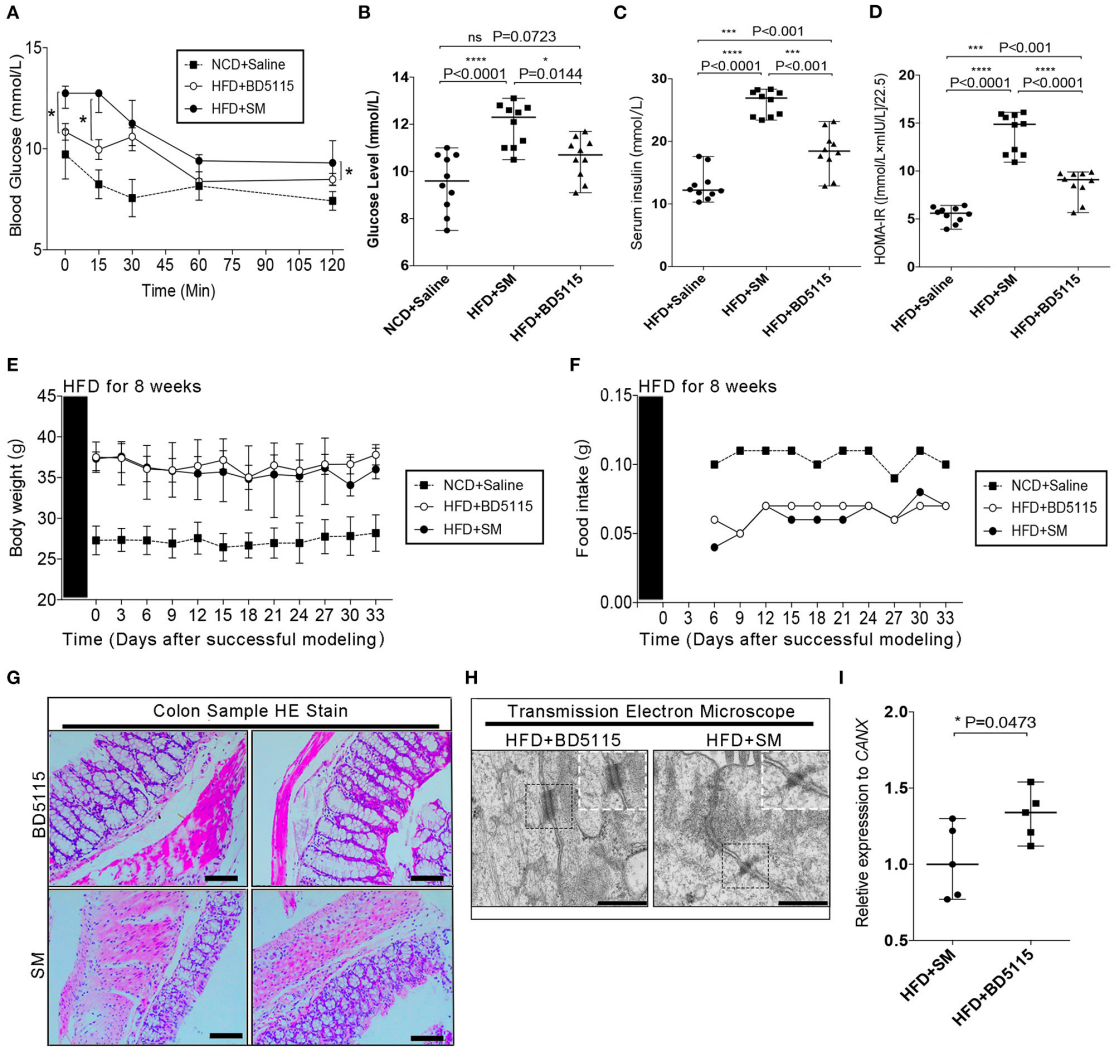
BD5115 metabolites ameliorate HFD-induced hyperglycemia. **(A)** Insulin tolerance test results from 19-week-old BD5115 and SM mice (*n* = 6 biologically independent animals per group, *: *P* < 0.05). Since each group has data at 5 different time points, we use the two-way ANOVA with Bonferroni *post-hoc* test method to compare between SM group and BD5115 group. **(B)** Fasting blood glucose levels in 19-week-old BD5115 and SM mice at 6 h after fasting (*n* = 10 biologically independent animals per group, the data are presented as the median with range, n.s.: non-significant, *: *P* < 0.05, ****: *P* < 0.0001, one-way ANOVA analysis with Bonferroni *post-hoc* test. *n* = 10 for NCD+Saline are also shown). **(C)** Serum insulin levels in 15-week-old BD5115 and SM mice at 6 h after fasting (*n* = 10 vs. 10 mice per group, respectively; the data are presented as the median with range, ***: *P* < 0.001, ***: *P* < 0.001, one-way ANOVA analysis with Bonferroni post hoc test. *N* = 10 for NCD+Saline are also shown). Statistical comparison was made between BD5115 group and SM group. **(D)** HOMA-IR values of 20-week-old mice (*n* = 10 for biologically independent BD5115 animals vs. *n* = 10 for SM animals; the data are presented as the median with range, ***: *P* < 0.001, ***: *P* < 0.001, one-way ANOVA analysis with Bonferroni *post-hoc* test. n=10 for NCD+Saline are also shown). **(E)** Diagram of changes in mouse body weight. The data are presented as the median with range. The x-axis shows the data after the start of intragastric administration of metabolites. We compared the data at 12 different time points of SM group and BD5115 group. **(F)** Diagram of changes in mouse food intake. The x-axis shows the data after the start of intragastric administration of metabolites. We compared the data at 10 different time points of SM group and BD5115 group. **(G)** H&E staining of colon tissue. The bar represents 100 μM. **(H)** Representative electron microscopy images of freshly collected colon tissues from BD5115 and SM mice. The bar represents 5 μm. **(I)** Changes in the expression of the *OCLN* were detected by qPCR (*n* = 5 for biologically independent BD5115 animals vs. *n* = 5 for SM animals; the data are presented as the Mean ± SEM, *P ≤ 0.05, Student's *t-*test).

Mouse models with impaired intestinal barrier can generally be induced with Dextran sodium sulfate (DSS), Lipopolysaccharide (LPS) and HFD. To further study the biological function of *L. paracasei* BD5115 metabolites *in vivo*, we first constructed a high-fat diet (HFD) C57BL/6J mouse model. Wild-type mice fed a 60% fat diet can exhibit not only a damaged gut barrier but also increased inflammatory responses *in vivo* and a reduced sensitivity of insulin receptors to insulin, which are hyperglycemic symptoms (24). Here, we used 10% skimmed milk as the control group (SM group), and the metabolites of *L. paracasei* BD5115 were administered via gavage at the standard dosage of 0.375 g/kg/d. Because HFD can lead to hyperglycemia, we first detect blood glucose-related indicators. The insulin tolerance test (ITT) and serum insulin detection were carried out after 4 weeks of gavage of metabolites. In the ITT, the fasting blood glucose level of the BD5115 group was significantly lower than that of the SM group (*P* < 0.05) (Figures 2A,B). After the intraperitoneal injection of insulin, the blood glucose levels of mice in the BD5115 group were significantly lower those of mice in the SM group at four time points, indicating that the glucose metabolism ability of mice in the BD5115 group was stronger. The serum insulin level in the BD5115 group was also significantly lower than that in the SM group (*P* < 0.01) (Figure 2C). Statistical analysis of the insulin resistance index (HOMA-IR) in the two groups of mice confirmed that the insulin resistance of mice in the BD5115 group was significantly relieved (*P* < 0.05) (Figure 2D). However, there was no significant difference between the body weight and food intake of the mice in the BD5115 group and the HFD group (Figures 2E,F).

Food is first absorbed in the intestine to enter the blood circulation. In previous studies, BD5115 metabolites were thought to regulate the gene expression in Caco-2 cells derived from colon tissue. Therefore, we hypothesized that BD5115 metabolites can affect intestinal cell proliferation. Thus, we observed microscopic changes in mouse colonic tissue by H&E staining and transmission electron microscopy. H&E staining revealed that the intestinal epithelial tissue in the BD5115 group was thicker than that in the SM group (*P* < 0.05) (Figure 2G). Transmission electron microscopy (TEM) revealed that the adhesion between some IECs in the BD5115 group was more regular and complete than that in the SM group (Figure 2H). Furthermore, in our qPCR experiments, we also confirmed that the expression of *Occludin* (*OCLN*), an important structural components of the tight junction, which are involved in the biogenesis and functional integrity of the intestinal barrier (25, 26), was increased in the colon tissue of the BD5115 group (*P* < 0.05) (Figure 2I). Based on these data, we suspect that metabolites may have the potential function of increasing the integrity of intestinal barrier by affect intestinal cell proliferation.

### Effects of BD5115 Metabolites on the Gene Expression and Types of Colon Cells as Determined by Single-Cell RNA Sequencing Analysis

Although BD5115 metabolites promote the proliferation of IECs in mice, the specific molecular mechanisms are not well understood. Here, mRNA sequencing was performed in colon tissues of mice fed a HFD and administered BD5115 metabolites via gavage. The mRNA sequencing results showed that the expression of the *MYC* gene was increased significantly in the BD5115 group (Supplementary Figure S2). Considering that *MYC* is a very important proto-oncogene involved in cell proliferation, its increased expression indicates decreased cell apoptosis and improved cell proliferation. Because these genes are expressed in the vast majority of cells, their expression in cells of other intestinal structures may have influenced the RNA sequencing results. Therefore, to obtain a detailed understanding of the mechanism by which BD5115 metabolites regulate colon tissue cells, we also performed single-cell RNA sequencing on colon tissues of the BD5115 and SM group.

**Figure 3:**
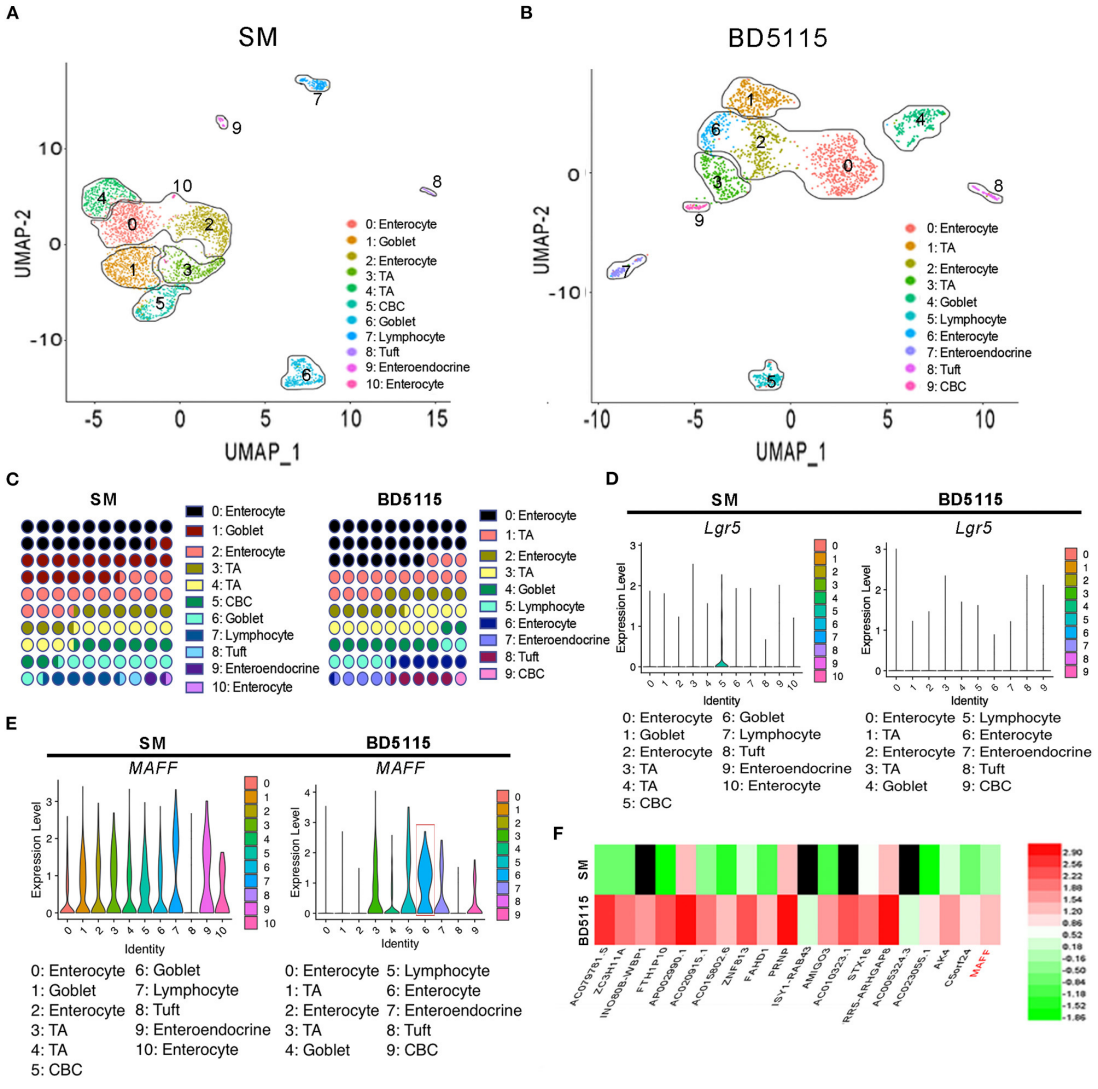
Single-cell RNA sequencing of colon tissues. UMAP analyses of colon tissues (*n* = 5 for biologically independent BD5115 animals vs. *n* = 5 for SM animals) from the SM group and BD5115 group are shown in **(A,B)**, respectively. UMAP clustering of all cells in the BD5115 and SM group after integration. Each color represent cells with a different sub-identity. **(C)** The proportions of different cell types in the colon tissues of mice in the SM group and BD5115 group. **(D)** *LGR5* gene expression in the SM group and BD5115 group. **(E)** *MAFF* gene expression in the SM group and BD5115 group. **(F)** Heatmap showing the DEGs in Caco-2 cells treated with BD5115 metabolites.

In the SM group, UMAP analysis revealed 11 different cell subtypes that were clustered. Accordingly, there were 10 cell subtypes in the BD5115 group (Figures 3A,B). They included IECs, transient proliferating cells, crypt-base columnar cells (CBCs), goblet cells, tuft cells, intestinal secretory cells, and CD8^+^ lymphocytes. The marker genes of the cell types are shown in Supplementary Table S2. Clusters 0, 2, and 10 in the SM group and clusters 0, 2, and 6 in the BD5115 group were IECs. Statistical analysis of the cell subtype numbers in the two groups showed that the proportion of IECs in the whole tissues of the BD5115 group was increased (Figure 3C). At the same time, the *LGR*5^+^ CBCs content was decreased significantly (Figure 3D). We hypothesized that BD5115 metabolites promote the proliferation of IECs. CBCs migrate from the base of the crypt to the surface and differentiate into IECs. As a negative feedback regulator, *LGR*5^+^ CBCs were depleted.

To determine why BD5115 metabolites promoted the increase in *MYC* gene expression in IECs, we analyzed the marker genes of IECs in the SM group and BD5115 group. We found a gene encoding a bZIP transcription factor, MAF bZIP transcription factor F (MAFF). This gene was specifically expressed in cluster 6 (IECs) of the BD5115 group (Figure 3E). Among all the cells of cluster 6, 68.1% expressed the *MAFF* gene. Correspondingly, only 17.7% of the cells in the other clusters expressed this gene. The mRNA sequencing of colon samples also showed that the *MAFF* expression was significantly increased in the BD5115 group (Supplementary Figure S2). Moreover, mRNA sequencing of Caco-2 cells treated with BD5115 metabolites showed that the *MAFF* gene expression was increased by approximately 4-fold (Figure 3F). Among all the genes with higher expression, *MAFF* was ranked 20th. This suggests that BD5115 metabolites may have similar molecular mechanisms to regulate *MAFF* expression.

### MAFF Directly Binds to the *MYC* Promoter and Interacts With MBP1 to Promote *MYC* Gene Expression

**Figure 4:**
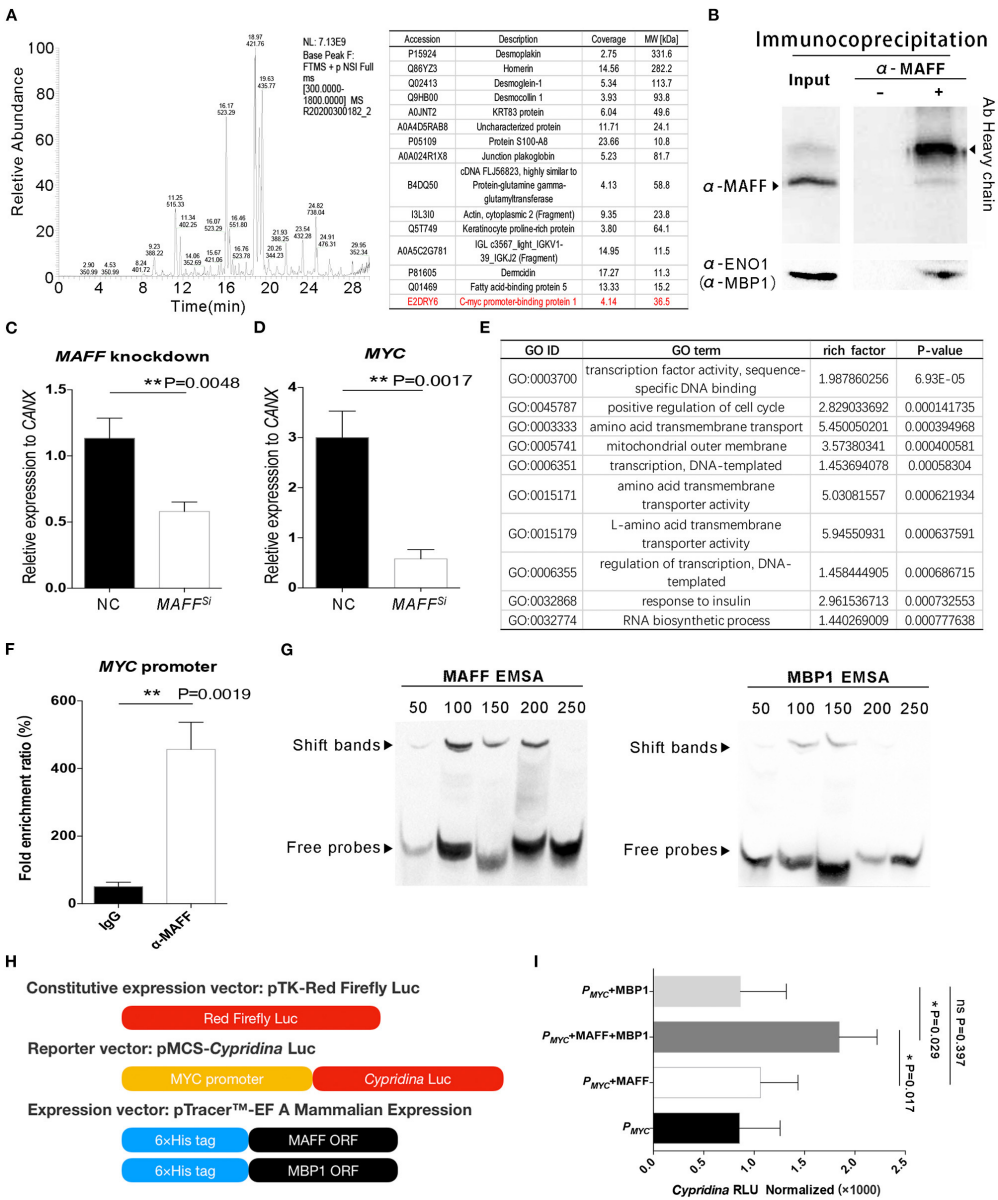
MAFF regulates *MYC* gene expression by interacting with MBP1. **(A)** Screening of MAFF-interacting proteins. The MAFF protein complex was captured by a MAFF antibody and analyzed by mass spectrometry. **(B)** Study of the interaction between MAFF and MBP1 by immunoprecipitation. Caco-2 cells were extracted with the MAFF antibody and purified by Protein-A agarose beads. Total protein and the protein precipitated by the MAFF antibody were cultured with MAFF and ENO1(MBP1) antibodies, independently. The heavy chain of MAFF antibody is also shown. **(C)** The siRNA fragment of *MAFF* was transfected into Caco-2 cells. After 24 h, the *MAFF* gene was detected by qPCR (*n* = 3 for biologically duplication, the data are presented as the mean ± SEM, **: *P* < 0.01, Student's *t-*test). **(D)** Verification of the difference in *MYC* gene expression by qPCR (*n* = 3 for biologically duplication, the data are presented as the mean ± SEM, **: *P* < 0.01, Student's *t-*test). **(E)** GO analysis was performed by RNA sequencing after MAFF knockdown. **(F)** Investigation of the interaction between MAFF and the *MYC* gene promoter by ChIP. Caco-2 cells were extracted with an MAFF antibody and purified by Protein-A agarose beads. Total DNA and DNA immunopurified by the MAFF antibody were detected by qPCR (*n* = 3 for biologically duplication, the data are presented as the mean ± SEM, **: *P* < 0.01, Student's *t*-test). **(G)** EMSA of MAFF and MBP1 binding to the *MYC* promoter *in vitro*. The upper black arrow identities the shifted bands due to the protein/DNA complex. **(H)** Schematic diagram of construction of experimental vector for transcription activation. **(I)** *Cypridina*/Firefly LUC indicates the ratio of *Cypridina* activity to firefly luciferase activity (*n* = 6, the data are presented as the mean ± SEM, n.s.: nonsignificant, *: *P* < 0.05, one-way ANOVA analysis with Bonferroni *post-hoc* test.).

The *MAFF* gene is located on chromosome 22 of the human genome and encodes a protein consists of 164 amino acids. Unlike traditional transcription factors, MAFF has no transcriptional activation domain. This means that MAFF must interact with other proteins to initiate transcription. Therefore, we first enriched the MAFF protein complex by immunoprecipitation. Then, the protein complex was analyzed by SDS-PAGE and mass spectrometry. After two repeated experiments, we found that *c-myc* promoter binding protein 1 (MBP1) potentially interacts with MAFF *in vivo* (Figure 4A). The MBP1 protein is produced by the intronic splicing of the *ENO1* gene (27). Although ENO1 is an enolase, MBP1 is thought to bind to the promoter of the *MYC* gene and inhibit its expression (28). Interestingly, we also found that some desmosome junction proteins (Desmoplakin, Desmoglein-1, Desmocollin 1, Junction plakoglobin and Dermcidin) related to the adhesion of IECs may also interact with MAFF *in vivo* (Figure 4A). It has been reported that transcription factors often leave the nucleus and enter the cytoplasm to interact with and facilitate the transport of cytoplasmic proteins (29, 30). Therefore, we believe that the interaction between MAFF and desmosome junction protein is feasible. In this work, we revealed that the *MYC* gene potentially plays a role in the biological functions of BD5115 metabolites. Therefore, we used a co-immunoprecipitation method to verify the protein interaction between MAFF and MBP1. After the MAFF protein complex was enriched by a MAFF antibody, the protein complex was detected by Western blot with an ENO1 (MBP1) antibody (Figure 4B). The results showed that MBP1 was present in the MAFF protein complex.

Although we proved the interaction between MAFF and MBP1, we were unable to confirm the specific relationship between MAFF and *MYC*. Here, we first knocked down the *MAFF* gene in Caco-2 cells using siRNA and then extracted the total RNA for mRNA sequencing analysis. We selected qPCR to detect the content of MAFF. The results of the mRNA sequencing showed that the expression of *MAFF* was decreased by approximately 60%. The Western blot results showed that the protein level of MAFF was also decreased significantly (Figure 4C). Consistent with the previous results, the mRNA sequencing results showed that the *MYC* gene expression was also downregulated after the *MAFF* expression was decreased (Supplementary Table S3). The qPCR results also verified these results (Figure 4D). GO analysis of differentially expressed genes (DEGs) revealed that the DEGs were mainly involved in transcription factor activity and the cell cycle, among other processes (Figure 4E).

In addition to knockdown of the *MAFF* gene, we used the chromatin immunoprecipitation (ChIP) method to verify the direct interaction between MAFF and the *MYC* promoter. We used an anti-MAFF antibody to enrich the DNA fragment that directly binds to MAFF. We designed primers based on the 250 bp sequence of the *MYC* promoter and amplified the promoter fragment by qPCR. The results showed that the *MYC* promoter was significantly enriched in MAFF antibody-treated samples compared with the IgG negative control samples (Figure 4F). This result confirmed the direct interaction between MAFF and the *MYC* promoter. Since MBP1 was previously reported to bind to the *MYC* promoter, we next explored the specific binding sites of MAFF and MBP1 in the *MYC* promoter region with Electrophoretic mobility shift assay (EMSA) experiments. We fused MAFF and MBP1 to a 6 × His tag and induced their expression. In addition, we segmented the *MYC* promoter to a length of 50 bp to verify the specific positions at which MAFF and MBP1 bind to the *MYC* promoter *in vitro*. After MAFF was combined with five different biotin-labeled probes, several shifted bands in the first 200 bp range were observable (Figure 4G). In addition, MBP1 also showed multiple shifted bands within 150 bp of the promoter region. This means that the binding sites of MAFF and MBP1 on the *MYC* gene promoter are in close proximity and that both have multiple binding sites.

To elucidate the biological significance of *MYC* gene expression after MAFF and MBP1 interaction, we studied transcriptional activation in depth with a dual-fluorescent vector system. Here, we utilized the luciferase reporter gene system made by Thermo Fisher Scientific Pierce. Red Firefly Luc was used as the construct expression vector. A 300 bp *MYC* promoter sequence was inserted into the reporter vector (pMCS-*Cypridina*-LUC). In addition, the coding sequences of the MAFF and MBP1 genes were inserted into the pTricer^TM^ EF A Mammalian expression system and transiently expressed the fusion proteins with a 6 × His tag in Caco-2 cells (Figure 4H). Detection of the transcriptional activation ability revealed that the enzymatic activities in cells transfected with the MAFF or MBP1 alone were not significantly different from those in control group cells. The reporter gene downstream of the *MYC* promoter was activated only after the cotransfection of MAFF and MBP1 (Figure 4I). This means that MAFF is indeed a direct transcriptional activator of the *MYC* gene.

### Among BD5115 Metabolites, HMBA Inhibits the Apoptosis of Caco-2 Cells by Promoting *MYC* Gene Expression

**Figure 5:**
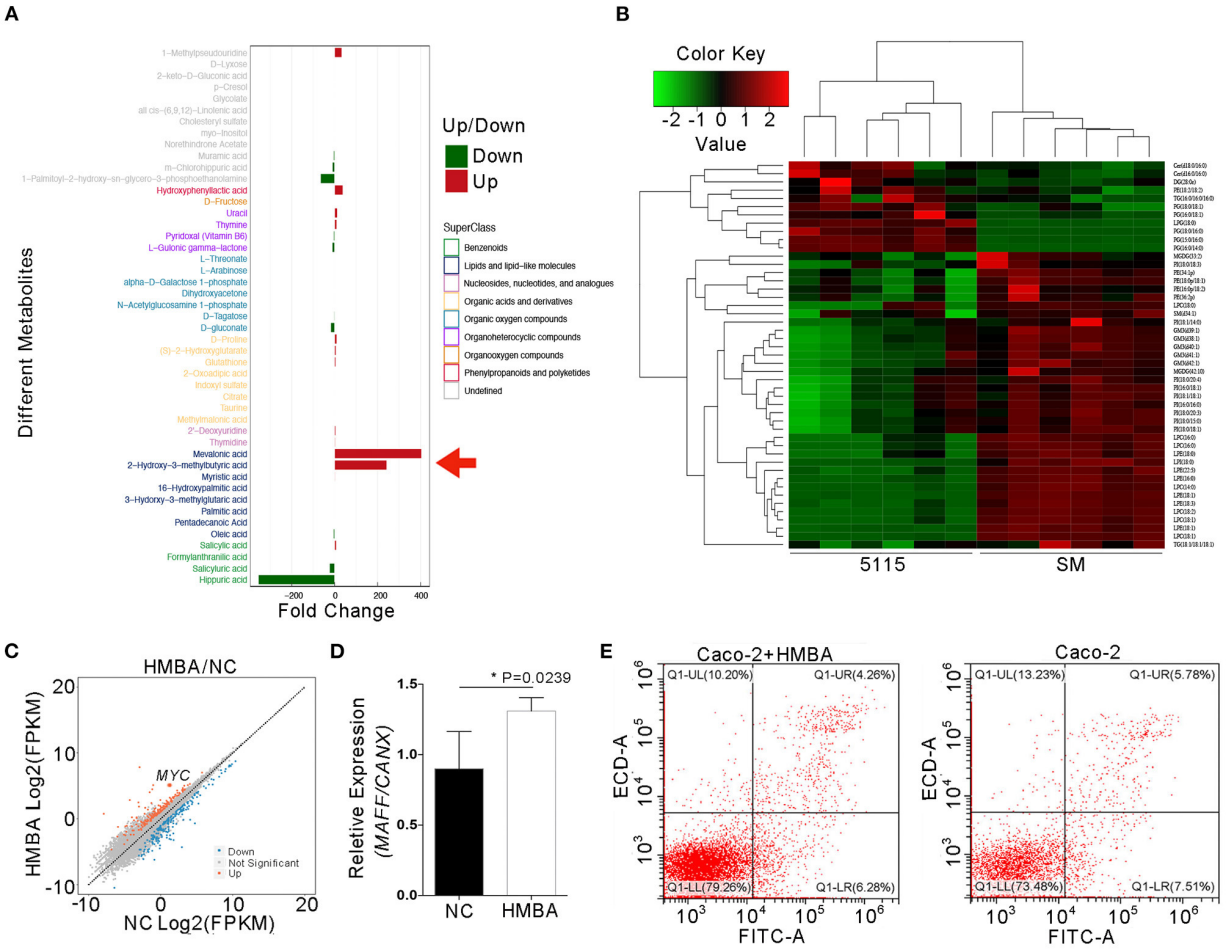
Identification of the active components of BD5115 metabolites. **(A)** The differences in BD5115 metabolites and skimmed milk as determined by metabonomics analysis. The red arrow represents the compound with significant increases in the BD5115 group. **(B)** Differences between BD5115 metabolites and skimmed milk in the liposome group. **(C)** Scatter plot of DEGs as determined by RNA sequencing. Caco-2 cells were treated with HMBA for 24 h, after which total RNA was extracted. **(D)** Verification of the *MYC* gene expression in Caco-2 cells treated with HMBA for 24 h by qPCR (*n* = 3 for biologically duplication, the data are presented as the mean ± SEM, * *P* < 0.05, Student's *t*-test). **(E)** The apoptosis of Caco-2 cells was analyzed by flow cytometry after *MYC* gene expression was increased.

To confirm the effective components of BD5115 metabolites in proliferation of IECs, the BD5115 metabolites were subjected to metabolome and liposome analyses. Metabolomic analysis revealed that the contents of mevalonic acid (MVA) and HMBA were increased significantly (200-400 fold) in BD5115 metabolites (Figure 5A, Supplementary Table S4). MVA is considered to be an important intracellular cholesterol metabolite that plays an important role in immunity and tumor proliferation (31, 32). Butyrate, a short-chain fatty acid, is also thought to play an important role in intestinal health (33). Liposome analysis revealed that the contents of ceramides and phosphatidylglycerol were slightly increased in BD5115 metabolites (Figure 5B). Considering that the contents of MVA and HMBA were significantly increased in BD5115 metabolites and that both have the potential to promote the proliferation of IECs, we hypothesized that these two substances are the active ingredients in BD5115 metabolites.

To verify this hypothesis, Caco-2 cells were treated with 50 mM MVA and HMBA. After 12-24 h, RNA was extracted for mRNA sequencing to analysis. In the HMBA-treated samples, *MYC* gene expression was increased by approximately 7-fold (Figure 5C and Supplementary Table S5). Meanwhile, we also found that the gene expression of its upstream regulator *MAFF* was increased by about 20%, while the expression of the *MYC* gene was significantly increased (Figure 5D). This suggests that although HMBA promotes *MAFF* expression, HMBA may promote *MYC* gene expression not only through MAFF itself. It can also promote *MYC* gene expression *in vivo* through a variety of signaling pathways. Because the *MYC* gene is related to cell proliferation, flow cytometry was performed on Caco-2 cells to analyze the effect of HMBA on the apoptotic ability of Caco-2 cells. The results showed that the apoptosis rate of untreated Caco-2 cells was 13.29% and that of HMBA-treated Caco-2 cells was 10.54% (Figure 5E). In conclusion, the above results confirmed that HMBA can inhibit the apoptosis and promote the proliferation of Caco-2 cells by upregulating the expression of the *MYC* gene.

### BD5115 Metabolites Do Not Promote Colitis-Associated Cancer (CAC) and Inhibit CAC Progression

**Figure 6:**
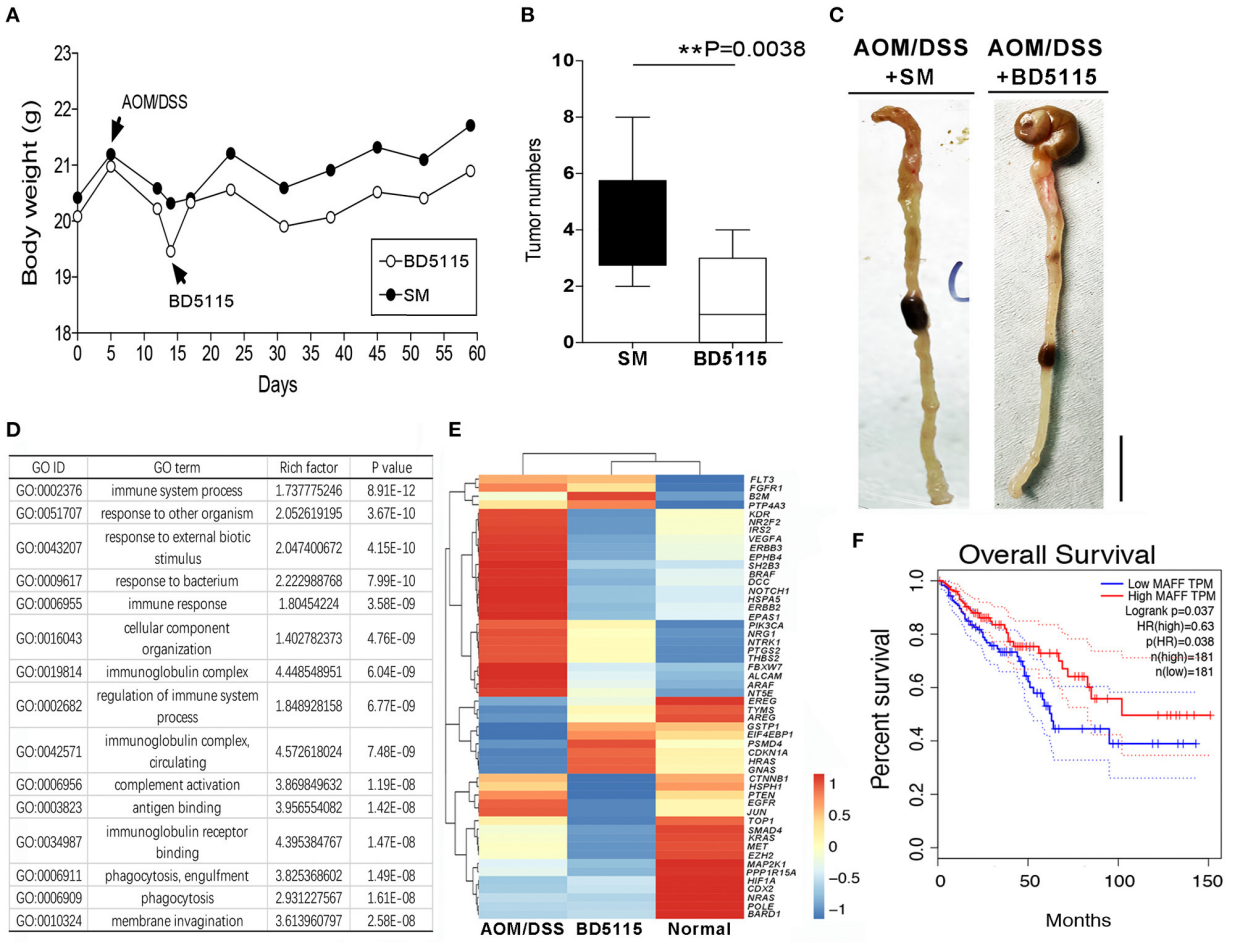
AOM/1.5% DSS induced CAC in mice. **(A)** Body weights of mice in the AOM/1.5% DSS group (*n* = 10 biologically independent animals per group). AOM (10 mg/kg) was intraperitoneally injected into 8-week-old mice on day 1, and 1.5% DSS was administered via the drinking water on day 5 for 5 d. **(B)** The numbers of colon tumors in BD5115 and SM group mice (*n* = 10 biologically independent BD5115 animals vs. *n* = 10 SM animals; ** *P* < 0.01, Student's *t*-test). **(C)** Macroscopic observation of the colon tissues of mice in the BD5115 group and SM group. The bar represents 1 cm. **(D)** GO analysis of differentially expressed genes in the distal colons of mice in the BD5115 group and SM group (*n* = 3 biologically independent BD5115 animals vs. *n* = 3 SM animals). **(E)** Heatmap of 53 CAC-related genes. **(F)** Survival curve analysis of the *MAFF* gene in the TCGA database.

Considering that the *MYC* gene is an important proto-oncogene, *MYC* mutation or overexpression drives the growth of numerous cancers. To evaluate the risk of tumorigenesis after the promotion of *MYC* gene expression by BD5115 metabolites, we established an AOM/1.5% DSS model in wild-type C57BL/6J mice. After AOM/1.5% DSS treatment, the weights of wild-type mice was decreased significantly, and BD5115 metabolites and skimmed milk were administered by gavage (Figure 6A). On the 60th day of the experiment, the mice were dissected to observe the numbers and morphologies of colon tumors. Our statistics analysis revealed that the tumor numbers in the colons of mice in the BD5115 group were not increased and actually decreased (*P* < 0.01) (Figure 6B). We also found that the intestinal tissues of mice in the BD5115 group were smoother than those of mice in the SM group (Figure 6C).

To further study the inhibitory effect of BD5115 metabolites on CAC at the molecular level, we harvested the distal colon tissues of mice, extracted RNA and performed mRNA sequencing. GO analysis showed that the BD5115 metabolites had the greatest effect on immune function in mice with CAC (Figure 6D). We then selected some genes related to CAC, such as *VEGFA, BRAF, Notch1, PIK3CA, NTRK1, PTEN*, and *EGFR*. The clustering results showed that the expression patterns of tumor-related genes in the BD5115 group were more similar to that in the normal group without treatment than in the CAC group (Figure 6E). This indicates that although we found that BD5115 metabolites can promote the expression of the *MYC* gene, the biological mechanisms by which they inhibit the development of CAC while promoting the proliferation of IECs remain unknown.

Moreover, analysis of the gene expression and survivability data for patients in the cancer genome atlas (TCGA) database revealed that the overall survival time of CAC patients with high MAFF gene expression was substantially longer than that of patients with low MAFF gene expression (p[HR]=0.038) (Figure 6F). These real-world data are consistent with our results and indicate that MAFF is a potential a marker of CAC.

## Discussion

**Figure 7:**
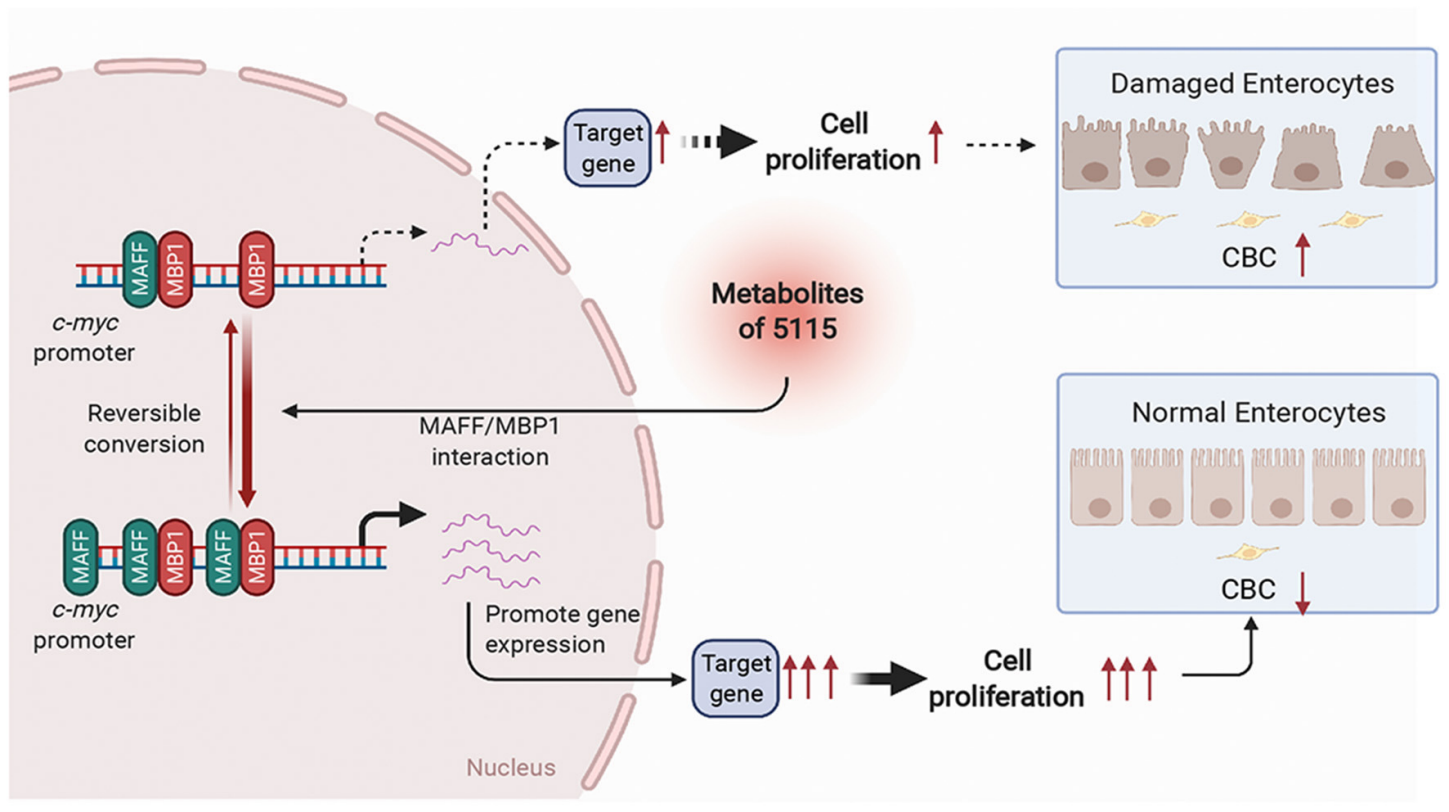
Schematic diagram of BD5115 metabolite-mediated *MYC* gene expression via the MAFF/MBP1 signaling pathway. The BD5115 metabolite promoted *MAFF* gene expression after entering cells. MAFF interacts with MBP1 and binds to the *MYC* promoter together. Since MBP1 suppresses the *MYC* gene, MAFF inhibits the function of MBP1 after interacting with MBP1. This allows the *MYC* gene to be heavily transcribed, thereby promoting the proliferation of IECs.

While probiotics are widely used as food additives, many studies on probiotics are limited to assessing efficacy in subjects and animal models. Different experiments adopt different recruitment standards and evaluation methods, often resulting in deviations of these efficacy evaluations (34). Therefore, it is very important to explore the molecular mechanisms by which probiotics regulate health function through molecular biology experiments. In this study, we screened a strain of *L. paracasei* BD5115. Molecular mechanism study found that its metabolites in IECs promoted the expression of *MYC* through MAFF/MBP1 pathway, thus promotes intestinal epithelial cell proliferation (Figure 7). This may promote the repair of intestinal barrier. This discovery is of great significance to the industrial and basic research uses of the BD5115 strain. Our findings not only advance our understanding of the mechanism by which BD5115 promote the proliferation of IECs but also reveal a new signaling pathway that regulates the expression of the *MYC* gene. In this pathway, we found that HMBA can significantly promote *MYC* expression in intestinal epithelial cells. As a metabolite of bacteria, although many studies have reported that short chain fatty acids are widely involved in the regulation of intestinal barrier, this work describes the specific biological functions of HMBA in detail. Correspondingly, recent studies have reported that butyrate can also reduce mitochondrial related cell death in intestinal inflammation and improve intestinal inflammation by inhibiting hexokinases 2 (HK2) (35). Therefore, the mechanism of butyrate regulating intestinal health may be multifaceted. The function of butyrate in regulating intestinal health needs to be further explored.

Considering that *MYC* is an important proto-oncogene, its overexpression or mutation plays a role in promoting the development of many kinds of tumors (36–39). Therefore, it is very important to discuss the consequences of *MYC* induction on the short and the long term. In the process of intestinal barrier damage, IECs tend to apoptosis and decrease significantly. This leads to continuous differentiation of CBCs (40, 41). In this process, the continuous proliferation of stem cells causes the accumulation of mutations on the chromosomes, which increases the incidence of tumors. For the *MYC* gene, although *MYC* overexpression increases the probability of tumor occurrence, the *MYC* gene itself plays an important role in cell proliferation and anti-apoptosis (42, 43). Therefore, when the intestinal barrier is impaired, the increase in *MYC* activity obviously contributes to the proliferation of IECs in the short term.

In a longer period of time, although there is a certain risk of increased *MYC* gene expression (44), we also believe that BD5115 metabolites have an inhibitory effect on colorectal cancer. To confirm this hypothesis, we conducted AOM/DSS mouse experiments. Unfortunately, although our RNA sequencing results confirmed that the immune functions in the colons of BD5115 group mice were significantly changed, the molecular mechanism by which BD5115 metabolites exert anticancer effects are still unknown. In our RNA sequencing data, we found significant changes in immune function in the colon in BD5115 group. This may be the reason why BD5115 metabolites exert anticancer effects. In addition, in our analysis of the TCGA database, we found that the high expression of *MAFF* significantly increased the survival rate of patients with CAC. Therefore, in the following studies, we believe that the study of the immune function of colon tissue and the *MAFF* gene will help to reveal the anticancer effects of BD5115 metabolites. In addition, although we confirmed that BD5115 metabolites have some antitumor properties, their effective components in the antitumor process need to be carefully elucidated. However, this may be very difficult due to the limitation of incomplete metabonomics databases. In fact, we found that HMBA was significantly enriched in BD5115 metabolites and strongly promoted the expression of the *MYC* gene in Caco-2 cells. However, its promotional effect on the expression of *MAFF* was weak. This means that the MAFF/MBP1 signaling pathway may not be the only one signaling pathway responsible for the regulation of *MYC* gene expression by HMBA. Some compounds other than HMBA among BD5115 metabolites may further promote the expression of the *MAFF* gene. Alternatively, the promotional effect of BD5115 metabolites on *MAFF* gene expression may be attributed to many compounds in combination. This is all to be further verified. Moreover, we also found that many immune-related signaling pathways have changed significantly in the AOM/DSS model. Because metabolites contain thousands of different compounds, its relationship with inflammation is not clear. In our research on the regulatory effects of mevalonate and HMBA on Caco-2 cells through RNA sequencing, we did not find significant changes in the signaling pathways related to inflammatory factors. However, we cannot therefore conclude that the metabolites of BD5115 have nothing to do with inflammation. Because, we only studied two compounds whose content in metabolites changed significantly. We are currently unable to determine whether other factors affect the occurrence of inflammation. But whether this is related to inflammation remains to be further studied. Finally, because we use the AOM/DSS model, which is an intestinal tumor model based on enteritis. In the real world, there are many inducing factors of colorectal cancer (45). The effects of metabolites on other types of colorectal cancer need to be further observed.

## Data Availability Statement

The datasets presented in this study can be found in online repositories. The names of the repository/repositories and accession number(s) can be found below: https://www.ncbi.nlm.nih.gov/, PRJNA702427, https://www.ncbi.nlm.nih.gov/, PRJNA722180.

## Ethics Statement

The use and care of the animals used in this research was reviewed and approved by the Shanghai Laboratory Animal Management Office (SYXK [Shanghai] 2017-0008). The animals used in the research were utilized based on appropriate experimental procedures. All of the animals were lawfully acquired, and their retention and use complied with federal, state and local laws and regulations in every case and were in accordance with the Institutional Animal Care and Use Committee of SLAC (IACUC) Guide for Care and Use of Laboratory Animals.

## Author Contributions

ZQ and WQ conceived and designed the experiments. ZQ, XW, CW, JH, HZ, ZL, and CY performed the experiments. ZQ analyzed the data and wrote the paper. All authors contributed to the article and approved the submitted version.

## Funding

This study was received funding from Bright Dairy & Food Co., Ltd. The funder was not involved in the study design, collection, analysis, or interpretation of data, the writing of this article or the decision to submit it for publication.

## Conflict of Interest

ZQ, XW, CW, JH, HZ, ZL, and CY are employed by Bright Dairy & Food Co., Ltd. The remaining author declares that the research was conducted in the absence of any commercial or financial relationships that could be construed as a potential conflict of interest.

## Publisher's Note

All claims expressed in this article are solely those of the authors and do not necessarily represent those of their affiliated organizations, or those of the publisher, the editors and the reviewers. Any product that may be evaluated in this article, or claim that may be made by its manufacturer, is not guaranteed or endorsed by the publisher.

## Supplementary Material

Supplemental Figure S1 PCA of the RNA sequencing results. Caco-2 cells were treated with 54 strains of bacteria and skimmed milk, and RNA extraction and sequencing were performed on all samples. Finally, a PCA map was constructed from the gene expression pattern of each sample.

Supplemental Figure S2 Scatter plot analysis of the RNA sequencing of colon tissues. Three mice in the BD5115 and SM group were randomly selected for RNA sequencing to analyze the DEGs. Scatter plot showing the upregulated and downregulated genes. The *MYC* and *MAFF* genes are shown in the Figure.

Supplemental Table S1 All information regarding the strains used in our screening. The information includes the species names and the IDs in our library.

Supplemental Table S2 Marker genes used for manual annotation. Marker genes used to identify the cell properties of each cluster were all listed in the table.

Supplemental Table S3 *MAFF* and *MYC* gene expression in NC group and siRNA group.

Supplemental Table S4 Metabonomics analysis of BD5115 metabolites. The table shows all the results of metabolomic analysis of BD5115 metabolites. The difference multiples in the table indicate the relative amounts of metabolites between BD5115 group and SM group.

Supplemental Table S5 *MAFF* and *MYC* gene expression in NC group and HMBA group.

Supplemental Table S6 Primer sequences in this study.

Supplemental Table S7 Probes used for EMSA.
