# Supplementary figures and images for: Lactobacillus paracasei BD5115-Derived 2-Hydroxy-3-Methylbutyric Acid Promotes Intestinal Epithelial Cells Proliferation by Upregulating the MYC Signaling Pathway
Source: Front Nutr. 2022 Mar 17;9:799053. doi: 10.3389/fnut.2022.799053 (PMC8968858; doi:10.3389/fnut.2022.799053)

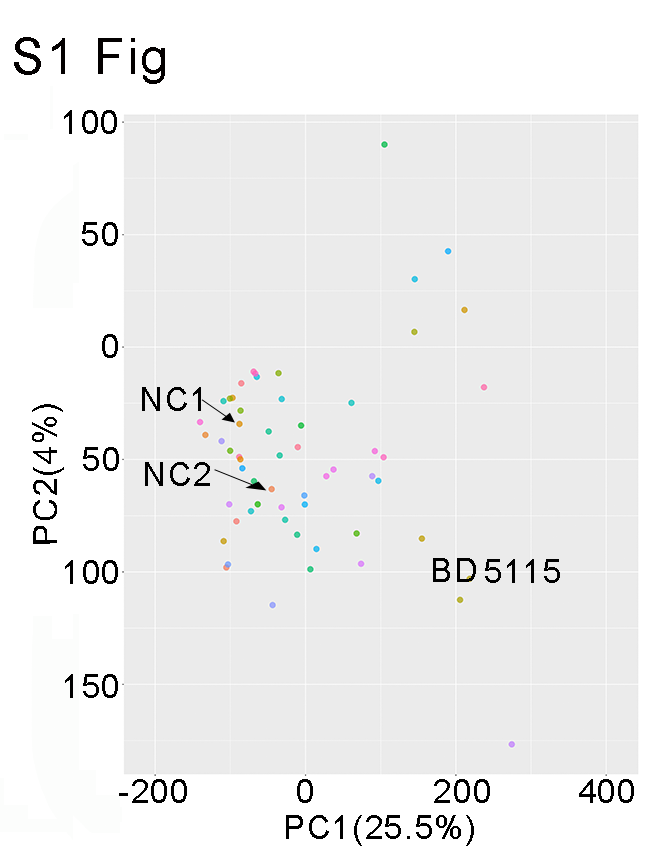

Supplement: Supplemental Figure S1 — PCA of the RNA sequencing results. Caco-2 cells were treated with 54 strains of bacteria and skimmed milk, and RNA extraction and sequencing were performed on all samples. Finally, a PCA map was constructed from the gene expression pattern of each sample. [file Image_1.TIF]

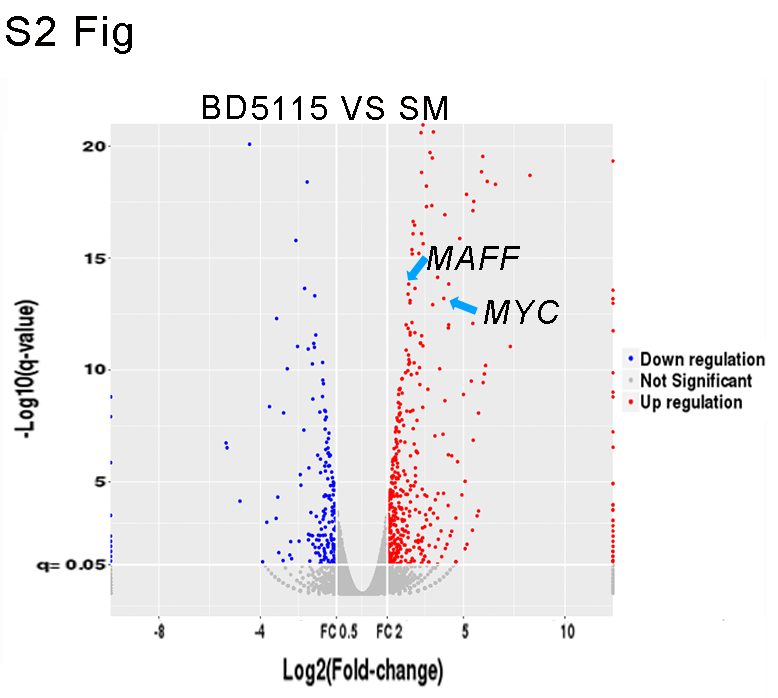

Supplement: Supplemental Figure S2 — Scatter plot analysis of the RNA sequencing of colon tissues. Three mice in the BD5115 and SM group were randomly selected for RNA sequencing to analyze the DEGs. Scatter plot showing the upregulated and downregulated genes. The MYC and MAFF genes are shown in the Figure. [file Image_2.TIF]
